# Metabolic Imaging of Human Kidney Triglyceride Content: Reproducibility of Proton Magnetic Resonance Spectroscopy

**DOI:** 10.1371/journal.pone.0062209

**Published:** 2013-04-19

**Authors:** Sebastiaan Hammer, Aiko P. J. de Vries, Paul de Heer, Maurice B. Bizino, Ron Wolterbeek, Ton J. Rabelink, Joost Doornbos, Hildo J. Lamb

**Affiliations:** 1 Department of Radiology, Leiden University Medical Center (Leiden University), Leiden, The Netherlands; 2 Department of Nephrology, Leiden University Medical Center, Leiden, The Netherlands; 3 Department of Endocrinology, Leiden University Medical Center, Leiden, The Netherlands; 4 Department of Medical Statistics, Leiden University Medical Center, Leiden, The Netherlands; University of Illinois at Chicago, United States of America

## Abstract

**Objective:**

To assess the feasibility of renal proton magnetic resonance spectroscopy for quantification of triglyceride content and to compare spectral quality and reproducibility without and with respiratory motion compensation in vivo.

**Materials and Methods:**

The Institutional Review Board of our institution approved the study protocol, and written informed consent was obtained. After technical optimization, a total of 20 healthy volunteers underwent renal proton magnetic resonance spectroscopy of the renal cortex both without and with respiratory motion compensation and volume tracking. After the first session the subjects were repositioned and the protocol was repeated to assess reproducibility. Spectral quality (linewidth of the water signal) and triglyceride content were quantified. Bland-Altman analyses and a test by Pitman were performed.

**Results:**

Linewidth changed from 11.5±0.4 Hz to 10.7±0.4 Hz (all data pooled, p<0.05), without and with respiratory motion compensation respectively. Mean % triglyceride content in the first and second session without respiratory motion compensation were respectively 0.58±0.12% and 0.51±0.14% (P = NS). Mean % triglyceride content in the first and second session with respiratory motion compensation were respectively 0.44±0.10% and 0.43±0.10% (P = NS between sessions and P = NS compared to measurements with respiratory motion compensation). Bland-Altman analyses showed narrower limits of agreement and a significant difference in the correlated variances (correlation of −0.59, P<0.05).

**Conclusion:**

Metabolic imaging of the human kidney using renal proton magnetic resonance spectroscopy is a feasible tool to assess cortical triglyceride content in humans in vivo and the use of respiratory motion compensation significantly improves spectral quality and reproducibility. Therefore, respiratory motion compensation seems a necessity for metabolic imaging of renal triglyceride content in vivo.

## Introduction

The global increase in chronic kidney disease (CKD) parallels the obesity epidemic [1]. Obese subjects have a weight-dependent 2- to 7-fold increased risk in progression of CKD irrespective of the underlying cause [2], [3]. Surprisingly, little is known on how obesity can progress or even lead to renal disease. Obesity is associated with a secondary form of focal segmental glomerulosclerosis, coined obesity-related glomerulopathy (ORG) when no other primary renal disease appears present. A recent study found a ten-fold increased incidence of ORG in native kidney biopsies between 1986–2000, suggesting a newly emerging epidemic [4]. Accumulating evidence from experimental and scarce limited human biopsy studies link ectopic lipid deposition in the kidney (*renal adiposity* or *fatty kidney*) to the development of ORG [5]–[8]. Unfortunately, clinical research on renal adiposity is hampered by the fact that it is considered unethical to biopsy human kidneys (repeatedly) without clear evidence of (late) renal disease.

Proton magnetic resonance spectroscopy (^1^H-MRS) is a tool for metabolic imaging to non-invasively assess triglyceride (TG) content in different human tissues in vivo, including the heart, liver and skeletal muscle [9], [10]. Spectral quality and reproducibility of e.g. cardiac ^1^H-MRS are influenced by respiratory motion [11]. Compensation for respiratory motion using navigator gating and volume tracking improves myocardial spectral quality and reproducibility [11], [12]. However, ^1^H-MRS for detection of renal TG content has never been performed to our knowledge. Furthermore, the possible effects of respiratory motion on reproducibility are unknown.

The purpose of this study was to assess the feasibility of renal ^1^H-MRS and to compare spectral quality and reproducibility without and with respiratory motion compensation in vivo. The results show that ^1^H-MRS is a feasible tool to assess cortical TG content in humans in vivo and use of respiratory motion compensation with navigator echoes significantly improves spectral quality and reproducibility.

## Materials and Methods

### Ethics statement

The Institutional Review Board of our institution (Leiden University Medical Center) approved the study protocol, and written informed consent was obtained from al participants. The research was conducted according to the principles expressed in the Declaration of Helsinki.

### Study design

A total of 37 healthy volunteers without a history of renal or cardiovascular disease, were prospectively included in the study. ^1^H-MRS of the renal cortex was performed at rest. ^1^H-MRS with and without respiratory motion compensation and volume tracking was performed during one session with the same parameters, without changing the position of the voxel. Thereafter, the volunteer was removed from the scanner. The volunteer was repositioned in the scanner and planning and ^1^H-MRS was repeated with and without respiratory navigator gating and volume tracking. The coil position was not marked.

### MR Technique

**Figure 1:**
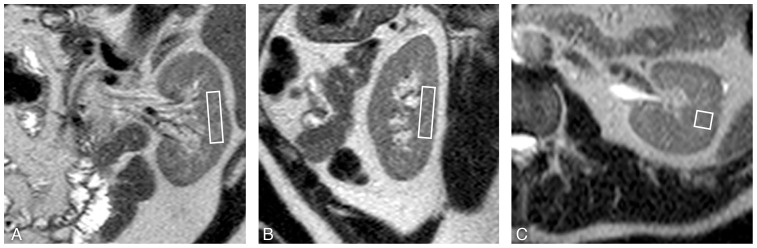
Planning of the proton magnetic resonance spectroscopy voxel. Placement of the proton magnetic resonance spectroscopy (^1^H-MRS) voxel (40×10×10 mm), avoiding contamination with intra-abdominal/perirenal and renal sinus fat. Coronal (A), sagittal (B) and transverse (C) scout images (single shot turbo spin echo).

Measurements were performed at 1.5T (Gyroscan ACS/NT15; Philips, Best, the Netherlands, using a 17-cm diameter circular surface coil, centered at the level of the left kidney. Scout images of the left kidney were made in a coronal, sagittal and axial orientation to verify coil position, Figure 1 (single-shot TSE sequence, shortest TR, TE = 80 ms, slice thickness = 3 mm, no gap).

### ^1^H-MR Spectroscopic Technique

Renal ^1^H-MR spectra were obtained from the cortex of the left kidney. The built-in body coil was used for RF transmission and the surface coil was used for signal reception. A 4 ml voxel (40×10×10 mm [height×length×width]) was positioned in the cortex on the scout images avoiding contamination from perirenal and renal sinus fat (Figure 1). In a subset of volunteers spectra with deliberate voxel misplacement with localization in the renal sinus were also measured. A point resolved spectroscopy sequence was used to acquire single voxel MR spectroscopic data. Per acquisition 1024 data points were acquired using 1000 Hz spectral bandwidth and averaged over 64 acquisitions. A TR of 3000 ms was chosen to approach complete relaxation of the TG signals. For respiratory motion compensation a pencil beam navigator was positioned on the right hemi-diaphragm [12]–[14]. A two-dimensional spatially selective RF pulse for pencil beam-shaped excitation was used. A pencil beam of 25 mm diameter was selected. Respiratory navigator gated spectroscopic data were accepted during in a predefined acceptance window of 5mm diaphragm displacement in end-expiration. Residual motion was compensated with motion tracking.

Preparation phases, including F_0_ determination, gradient shimming and water suppression were performed using respiratory motion compensation. To generate an internal reference, a spectrum without water suppression with a repetition time of 10 s and 4 averages was obtained, without changing any other parameter. Total acquisition time for both a water suppressed and water unsuppressed spectrum, including (re)positioning of the patient, shimming, and parameter adjustment for water suppression, was on average 20 min.

### Spectral Quantification

^1^H-MR spectroscopic data were fitted in the time domain, directly on free-induction decays using Java-based MR user interface software (jMRUI version 4.0; developed by A. van den Boogaart, Katholieke Universiteit Leuven, Leuven, Belgium)[15]. Data were analyzed within consensus by two authors with 7 (S.H.) and 20 (H.L) years of experience. The Hankel-Lanczos filter (single-variable decomposition method) was used to remove residual water signal from spectra acquired with water suppression. Renal cortical TG signal amplitudes were analyzed automatically by using the frequency estimates for renal lipids, described with the assumption of Gaussian line shapes at 0.9, 1.3, and 2.1 ppm. Prior knowledge was incorporated into the fitting [16]–[18]. Fixed frequencies for TG peaks were used, line widths and amplitudes were unconstrained. The zero-order phase correction was estimated by using the AMARES algorithm. The water signal peak at 4.7 ppm was quantified and its linewidth was estimated by using a Lorentzian line shape in the AMARES algorithm. The percentage of myocardial TG relative to water (%TG) was calculated as area under TG peak/area under water peak ×100. Spectral quality was quantified as the full width at half maximum (FWHM) of the water resonance in the unsuppressed spectra with, and without respiratory motion compensation.

### Statistical Analysis

FWHM was quantified using a paired T-Test for all data pooled (linewidth). Mean coefficients of variations for both ungated and gated measurements were quantified separately. To compare reproducibility of renal %TG with and without respiratory navigator gating and volume tracking, the intra-class correlation coefficients (ICC, two-way mixed, absolute agreement) were calculated by using a mixed effects ANOVA (with patients as random factor) for both conditions separately. Moreover, Bland-Altman plots were constructed. A test by Pitman was performed to analyze whether the correlated variances of the two techniques differed [19]. P<0.05 was considered significant. Means±standard errors (SE) are displayed. Statistical analyses were performed by using SPSS, version 20.01.

## Results

### Participants

Since the start of the technique developmental phase, ^1^H-MRS of the kidney was performed prospectively in 37 consecutive healthy volunteers without a history of renal or cardiovascular disease. A total of 17 volunteers were excluded from analyses during this initial phase: A) due to technical optimization and time constraints the scanning could not be completed [9 subjects], B) erroneous navigator [2 subjects] and C) impossible fitting procedure of all acquired spectra [6 subjects]). Based on these initial scans, the optimal technical protocol was defined.

In 20 volunteers the complete scanning protocol was completed (12 males, mean age±SD 26.3±8.3 yrs, range 20.3 – 57.9 yrs). In 4 of these volunteers the spectra acquired without respiratory motion compensation were not used for analysis, due to their degraded quality.

The linewidth of the unsuppressed water signal changed from 11.5±0.4 Hz to 10.7±0.4 Hz (all data pooled, p<0.05), without and with respiratory navigator gating respectively.

**Figure 2:**
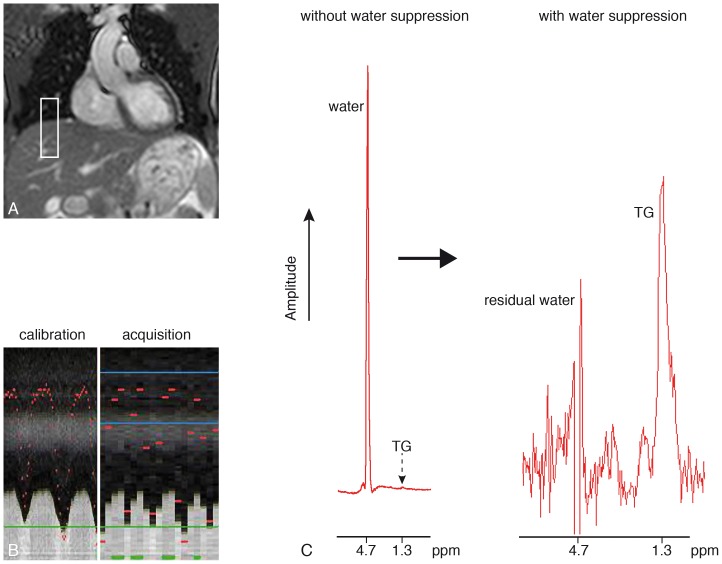
Planning of the respiratory navigator. The navigator pencil beam is positioned on the right hemidiaphragm (panel A). Panel B shows the calibration phase with a high temporal resolution, in which the lung-liver interface is detected during free breathing, as well as the acquisition phase showing the accepted measurements in the predefined end-expiratory window (lower temporal resolution). Panel C shows a typical non-suppressed (left) and a suppressed (right) localized renal proton spectrum, obtained using respiratory motion compensation and gating.

**Figure 3:**
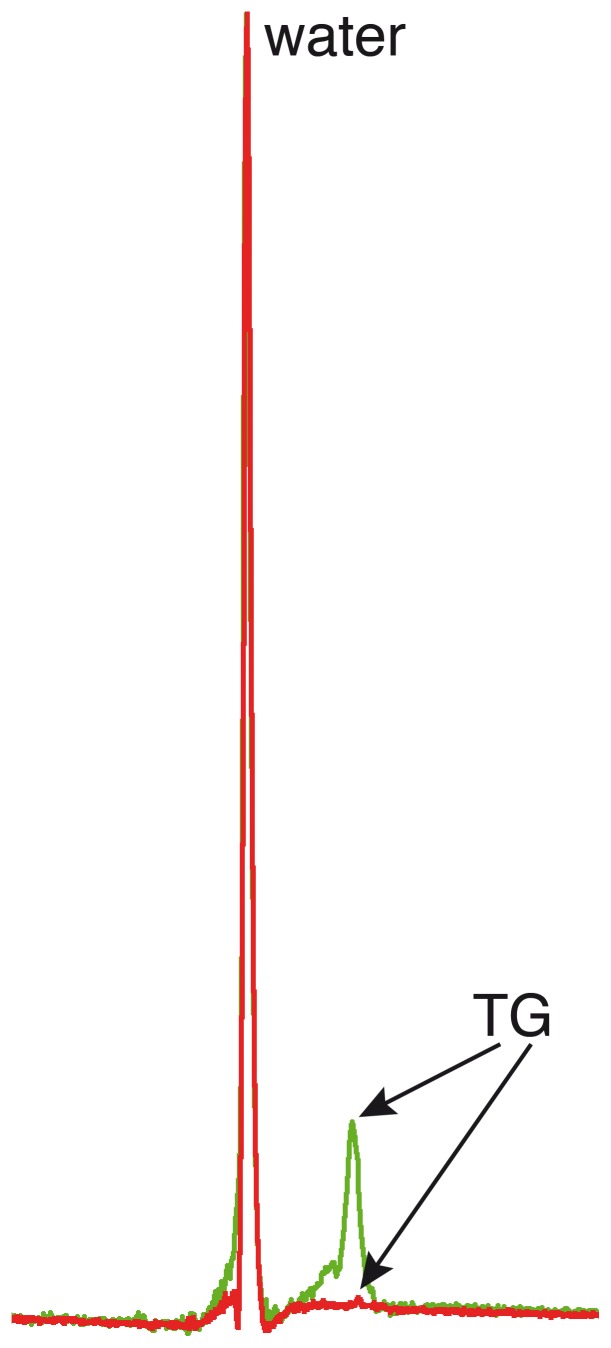
Examples of spectra including the renal parenchyma and sinus, and including only the renal parenchyma. Unsuppressed localized renal proton spectra of triglyceride (TG) content with deliberate planning of the voxel including the renal sinus (in green) and including only the renal parenchyma (in red). Percentages TG content in this particular volunteer were 18% (renal sinus) and 0.64% (parenchyma).

A typical example of a ^1^H-MR spectrum of renal cortical TG content is shown in Figure 2. An example of a spectrum obtained in the region of the renal sinus shows an expectedly high percentage of TG content (Figure 3).

Mean %TG in the first and second session without the use of the respiratory navigator were respectively 0.58±0.12% and 0.51±0.14% (P = NS). Mean %TG in the first and second session with respiratory navigator gating were respectively 0.44±0.10% and 0.43±0.10%. (P = NS between sessions and P = NS compared to respiratory gated measurements). One volunteer showed higher TG content compared to all others (2.1 and 2.2% with the use of respiratory motion compensation and without motion compensation respectively), albeit no technical explanation could be found (e.g. erroneous planning or navigator dysfunction).

**Figure 4:**
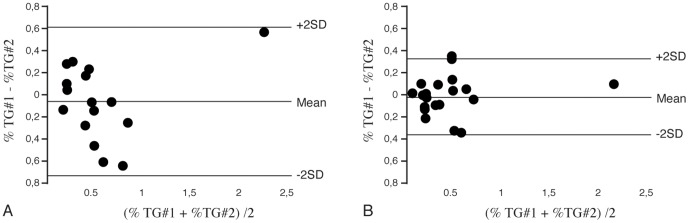
Reproducibility plots of renal triglyceride content. Bland-Altman plots of renal cortical triglyceride (TG) content calculated from spectra without (n = 16, A) the use of the respiratory navigator, and with (n = 20, B) the use of the respiratory navigator showing narrower limits of agreement/improved reproducibility.

Coefficient of variation without the use of the navigator was 42% which improved to 27% with use of respiratory motion compensation. ICC without the use of the respiratory navigator was 0.79 (95% confidence interval 0.51–0.92, P<0.001) and improved to 0.92 (95% confidence interval 0.81–0.97, P<0.001) with the use of respiratory motion compensation. Moreover, Bland-Altman plots (Figure 4) showed narrower limits of agreements with the use of the respiratory navigator, with a significant improvement in variability with the use of the respiratory navigator compared to ungated measurements, Pearson correlation (derived from the test by Pitman) of−0.59, P<0.05.

## Discussion

The present study shows that metabolic imaging of the human kidney for detection of cortical TG content using ^1^H-MRS is feasible. Respiratory motion compensation improves spectral quality and measurement reproducibility.

Intracellular lipid accumulation causing lipotoxicity in humans with the metabolic syndrome or type-2 diabetes mellitus, as assessed with ^1^H-MRS, has been associated with organ dysfunction, such as non-alcoholic fatty liver disease [20], cardiac diastolic dysfunction [9], [21] and pancreatic beta cell dysfunction [22], [23]. Moreover, respiratory motion compensation techniques to assess these lipid pools have been used in ^1^H-MRS previously, including the heart and pancreas [11], [24], [25]. The mean percentage of renal TG content in the present study content is in line with previous, Dixon-based techniques [26].

The ratio of TG and water show values which are comparable to the quantified amounts of TG found in the human heart [11]. However, one subject showed a consistently higher percentage, which could not be attributed to technical failure, physical activity or nutritional status. Moreover, this volunteer was not different from other subjects in terms of age. In contrast, deliberate planning of the voxel in the region of the renal sinus in a subset of volunteers showed clear overestimation, with TG values ranging from 8 to 25 percent. We can therefore not exclude a relatively wide range in physiological TG content in humans. Future studies on the effects of age, gender differences and the relation of renal cortical TG content with parameters of renal function or proteinuria should address this variability in TG content. A recent study indeed showed an age related increase in renal lipid content in mice, associated with increased glomerulosclerosis [27], as well as an increase after a high-fat diet [28]. Moreover, high-fat diet induced renal steatosis is reversed with the use of a peroxisome proliferator-activated alpha agonist (which regulates renal lipolysis) in mice, with associated improvements in albuminuria and fibrosis [29]. In addition to the PPAR-gamma pathway, the renin-angiotensin aldosteron system activation has also been linked to renal adipogenesis. Lisinopril, an ACE-inhibitor, caused normalization of renal adipogenesis and diminished lipid accumulation in uninephrectomized rats [30]. Moreover, it was shown in pigs with the metabolic syndrome that renal adiposity was associated with glomerular hyperfiltration and microvascular proliferation [6]. These animal studies suggest that renal adipogenesis is linked to critical pathways in obesity-associated renal disease with possibilities for intervention. However, data in humans is scarce. In humans renal sinus fat is associated with intra-abdominal and retroperitoneal fat content and with control of hypertension [31].

During respiration, movement of the diaphragm causes a displacement of the kidney relative to the spectroscopic voxel. Respiration may thereby influence spectral accuracy and reproducibility by influencing shimming, water suppression as well as varying partial volume changes in fat fraction. To overcome these problems, respiratory motion compensation has been used before to improve ^1^H-MRS spectral quality. For example by using triggering on exhalation [32], or with breath-hold acquisition [33]. Both studies were not primarily designed for detection and reproducibility of renal TG content. However, one study reports variations in lipid content as a consequence of contamination with lipids from outside the region of interest during free breathing. The present results are in line with these findings, as spectral quality and reproducibility of renal TG stores improved with respiratory motion compensation. Moreover, all spectra obtained with respiratory motion compensation could be accurately fitted, whereas 4 of the spectra obtained without respiratory motion compensation were of insufficient quality, due to poor water suppression or ghosting signals.

The improvements in spectral quality in terms of linewidth are in line with previous reported values for cardiac and skeletal muscle spectroscopy [11], [34].

Moreover, the use of the respiratory navigator improved the coefficient of variation and showed narrower limits of agreement in Bland-Altman analyses. However, the resulting coefficient of variation of 27% remains relatively high. We hypothesize this may be due to local inhomogeneities in renal parenchymal TG content, or variations in cortical and medullar TG content contribution within the voxel, as the voxel is close to the renal medulla and TG content may be distributed differentially [35], [36]. Nevertheless, reproducibility improved with respiratory motion compensation and seems necessary for human in vivo TG quantification.

A limitation of our study may be that ^1^H-MR spectra were obtained in one position in a single kidney only. Future studies should focus on possible regional differences in lipid content, possible differences in position in the cortex or medulla and the relationship with parameters of the metabolic syndrome, such as abdominal fat content, liver fat content and clinical parameters such as body mass index, serum TG content, cholesterol as well as renal function and proteinuria.

## Conclusions

Metabolic imaging of the human kidney using ^1^H-MRS is a feasible tool to assess cortical TG content in humans in vivo and the use of respiratory motion compensation with navigator echoes significantly improves spectral quality and reproducibility. Therefore, respiratory motion correction seems a necessity for metabolic imaging of renal TG content in vivo.
